# Human Lungs

**Published:** 1871-11

**Authors:** 


Human Lungs. — If every air cell in the lungs were cut open and spread out on a wall, they would cover a space of twelve yards each way; that is, at every full breath, the air drawn in is spread over a surface of a hundred and fifty yards.

